# The efficacy and potential predictive factors of PD-1/PD-L1 blockades in epithelial carcinoma patients: a systematic review and meta analysis

**DOI:** 10.18632/oncotarget.11291

**Published:** 2016-08-15

**Authors:** Yufan Yang, ZhaoFei Pang, Nan Ding, Wei Dong, Wei Ma, Yun Li, Jiajun Du, Qi Liu

**Affiliations:** ^1^ Institute of Oncology, Shandong Provincial Hospital Affiliated to Shandong University, Shandong University, Jinan, Shandong, Peoples's Republic of China; ^2^ Department of Thoracic Surgery, Shandong Provincial Hospital Affiliated to Shandong University, Shandong University, Jinan, Shandong, Peoples's Republic of China; ^3^ Department of Oncology, Shandong Provincial Hospital Affiliated to Shandong University, Shandong University, Jinan, Shandong, Peoples's Republic of China

**Keywords:** PD-1/PD-L1 blockades, epithelial carcinoma, predictive factor, outcome, immunotherapy

## Abstract

**Background:**

This systematic analysis aims to assess the efficacy of PD-1/PD-L1 blockades compared with non-PD-1/PD-L1 therapy and investigate the potential predictive factors in epithelial carcinoma patients.

**Results:**

A total of 11 trials with 6716 patients of melanoma, non-small cell lung cancer (NSCLC) and renal cell carcinoma (RCC) were included. The pooled HRs (95%CI) were 0.67 (0.62, 0.73), *p* < 0.001 for OS and 0.66 (0.57, 0.76), *p* < 0.001 for PFS. In subgroup analyses, HRs were 0.58 (0.50, 0.66), *p* < 0.001 in PD-L1 ≥ 1% group, 0.75 (0.63, 0.89), *p* = 0.001 in PD-L1 < 1% group for OS and 0.59 (0.48, 0.72), *p* < 0.001 in PD-L1 ≥ 1% group, 0.80 (0.59, 1.07), *p* = 0.136 in PD-L1 < 1% group for PFS. The *p* values of pooled HRs for OS in different age, sex and ECOG score groups were less than 0.001. In NSCLC patients, aggregated HRs for OS were 1.40 (0.92, 2.12), *p* = 0.114 in *EGFR* mutant group and 0.88 (0.59, 1.32), *p* = 0.536 in never smokers.

**Methods:**

A systematic search from January 2010 to April 2016 was conducted for eligible clinical trials. Based on the data of hazard ratios (HRs) and 95% confidence intervals (CIs) for overall survival (OS) and progression-free survival (PFS), we assessed the pooled HRs and proposed the subgroup analyses.

**Conclusions:**

PD-1/PD-L1 blockades prolonged OS and PFS in epithelial carcinoma patients. PD-L1 expression was a predictive factor for PFS but not predictive for OS. Age, sex and ECOG score were excluded to predict any of the efficacy endpoints. Smoking history and *EGFR* wild type were associated with extended OS in NSCLC patients.

## INTRODUCTION

The checkpoint immunotherapy has been increasingly understood and used to unleash the immune system to fight against cancer [1]. These years, several immune checkpoints including CTLA-4 and PD-1/PD-L1 were identified and multiple agents have been developed to bind with the immunologic checkpoints and block checkpoint-pathways, which would otherwise impair the T cell anti-tumor activity. The efficacy of those agents in promoting immune recognition, enhancing the immune response with T cell and reducing the immune tolerance of tumor development has aroused tremendous enthusiasm in cancer treatment nowadays [2].

As one of the most critical checkpoint immunologic treatments, PD-1/PD-L1 blockade has become a promising focus of immunotherapy in cancer treatment [3]. Two antibodies targeting PD-1: nivolumab (Opdivo, Bristol-Myers Squibb, a fully human monoclonal IgG4 antibody), pembrolizumab (Keytruda, Merck, a humanized monoclonal IgG4 antibody) and an antibody against PD-L1 named atezolizumab (Roche, a fully humanized, engineered monoclonal antibody of IgG1 isotype) have been approved by US Food and Drug Administration (FDA) [3, 4]. Moreover, at the present time of manuscript, the FDA has approved anti-PD-1/PD-L1 therapy for four histologic types of cancer: melanoma, non-small cell lung cancer (NSCLC), renal cell carcinoma (RCC) and metastatic urothelial carcinoma, all of which are epithelial carcinoma [5].

Almost each of PD-1/PD-L1 blockades has satisfying overall response rates in treating different types of epithelial carcinoma [6, 7]. However, the outcome of patients treated with PD-1/PD-L1 blockades is still undetermined, for results of several relevant trials show insignificant improvement in prolonging overall survival (OS) and progression free survival (PFS). Besides, it is still urgently necessary to determine which specific group of patients will benefit from the anti-PD-1/PD-L1 therapy. Most of the present systematic studies focus on the response rate and safety of PD-1/PD-L1 blockades or perform single-arm meta-analyses to evaluate the biomarkers. We conduct the systematic analyses with strictly selected randomized controlled trials to clarify the efficacy and factors indicating the outcome of anti-PD-1/PD-L1 treatment, compared to other controlled interventions within epithelial carcinoma patients by analyzing HRs for OS or PFS.

In consideration of predictive factors, we analyze different membranous PD-L1 expression levels, because PD-L1 expression of tumor cells is most abundant in epithelial carcinoma and most closely correlated with response to anti-PD-1/PD-L1 agents [5, 8, 9]. Besides, baseline characteristics including age, sex and Eastern Cooperative Oncology Group (ECOG) score are the candidate factors to explore and subgroup analyses of squamous cancer, smoking status, *EGFR* mutation (within NSCLC patients) and *BRAF* mutation (within melanoma patients) are also conducted to provide further evidence for clinical treatment.

## RESULTS

### Study identification

**Figure 1:**
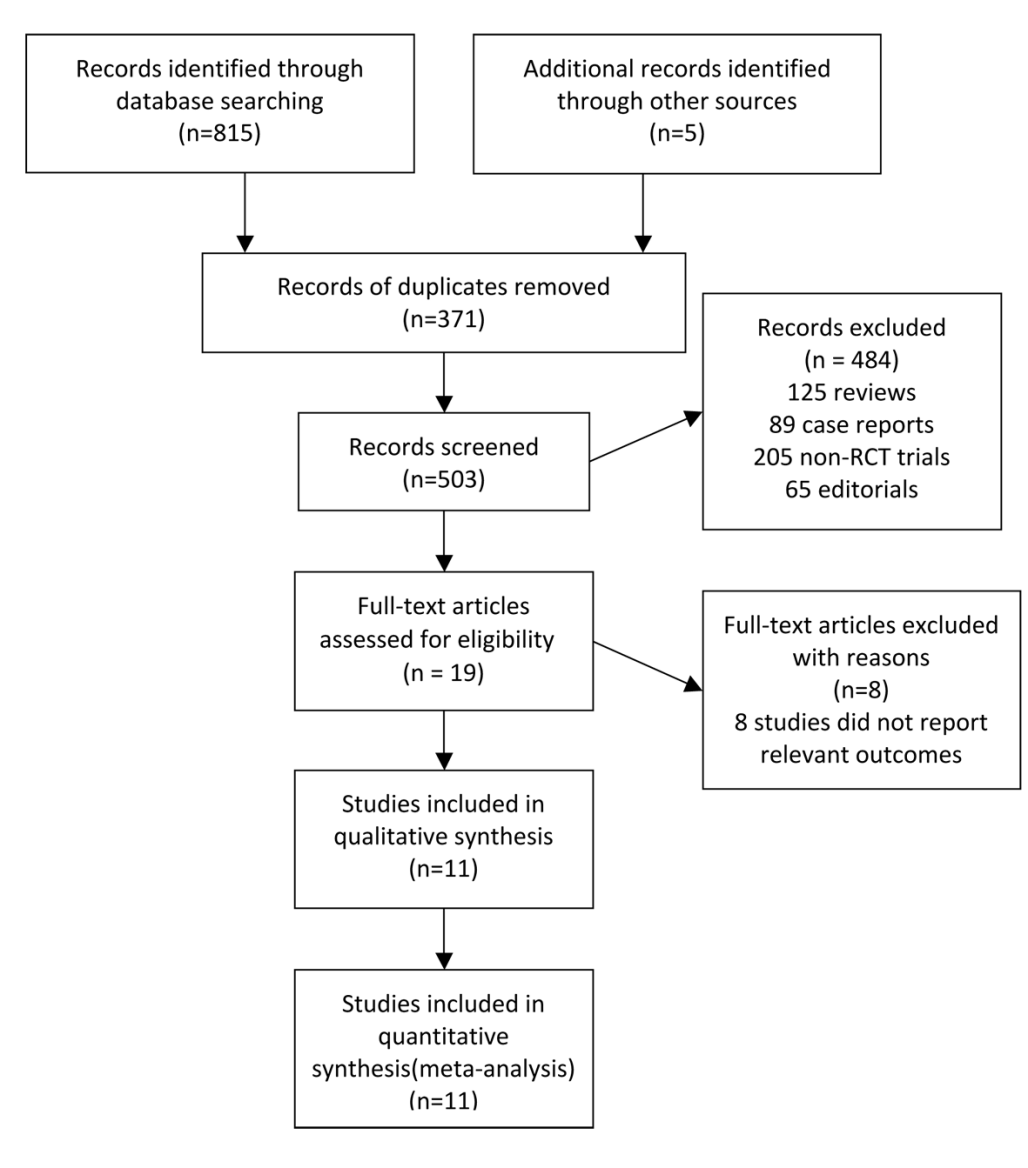
Flowchart of study selection procedure

**Figure 2:**
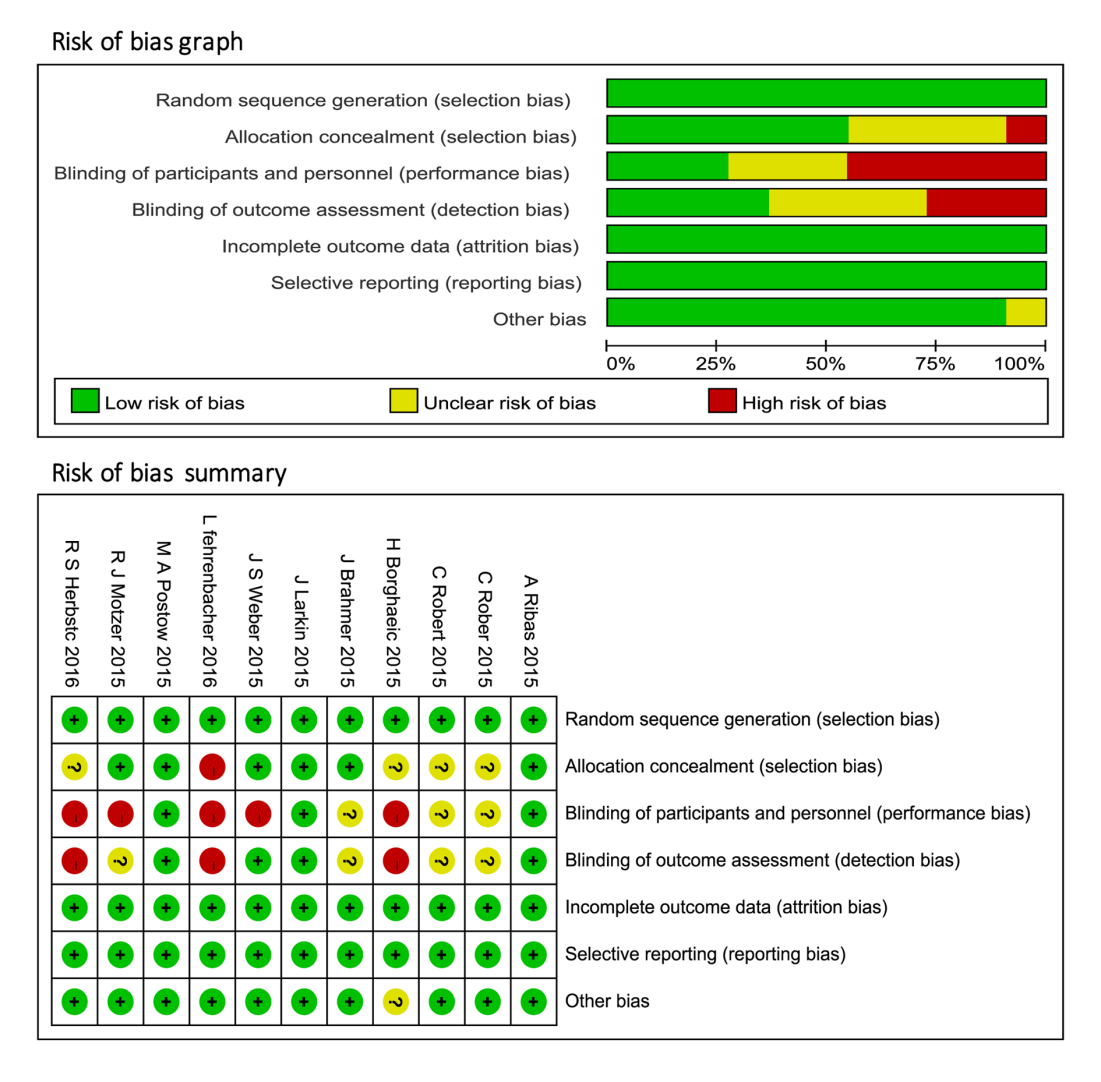
Risk of bias graph and summary of included clinical trials

According to the outlined search strategy, a total of 820 records were obtained, of which 371 duplicates were removed. After screening, 484 articles including reviews, case reports and non randomized controlled trials were excluded. Of the rest 19 records, 8 studies did not report the relevant data. Upon the remaining 11 studies, the two reviewers had the perfect agreement on their eligibility and assessed the quality of included studies independently by the scoring criteria stated in *Cochrane handbook for systematic reviews of interventions*. The study selection process was presented in Figure 1. The risk of bias graph and summary of selected studies generated by Revman.5.3 were showed in Figure 2.

### Characteristics of studies

The analyses were based on data from a total of 6716 patients enrolled in 11 randomized controlled trials. The experimental treatment drugs of those trials were PD-1/PD-L1 blockades, including nivolumab, pembrolizumab and atezolizumab, while the controlled interventions were standard chemotherapy (docetaxel, dacarbazine, etc), targeted therapeutic agents (everolimus) and other form of immunotherapy (ipilimumab). According to currently completed trials focused on epithelial carcinoma, all randomized controlled trials were conducted within melanoma, NSCLC and RCC patients. 6 of the enrolled trials were in melanoma patients (*n* = 3510), 4 in NSCLC patients (*n* = 2385), and 1 in RCC patients (*n* = 821). 3 of the trials were phase 2 trials, 1 was phase 2/3 trial, and 7 were phase 3 trials.

We collected the basic characteristics of patients in each included trial and extracted information to obtain hazard ratios (HRs) for OS and PFS of patients. For the PD-L1 expression evaluation, the immunohistochemistry assays of PD-L1 employed in the selected studies contained Dako, clone 28-8 (Epitomic) and 22C3 antibody (Merck). We retrieved the corresponding HR estimates with the cut-off of 1%, which meant membranous PD-L1 staining in at least 1% of tumor cells. The information of included studies' authors, cancer types, numbers of patients, interventions, basic characteristics of patients, and HRs for OS and PFS of PD-1/PD-L1 treatment *versus* non-PD-1/PD-L1 therapy were summarized in Table 1.

**Table 1:** The patients' characteristics and outcomes data of clinical trials included

First author	Year	Total	Intervention	Cancer type	Med age	Sex (male[%])	Harzard Ratio (95%CI)		Ref
							OS	PFS	
J Brahmer	2015	272	nivolumab 3mg/kg 2wk	NSCLC	62	82	0.59	0.62	[23]
			docetaxel 75 mg/m^2^ 3wk		64	71	(0.44, 0.79)	(0.47,0.81)	
H. Borghaei	2015	792	nivolumab 3mg/kg 2wk	NSCLC	61	52	0.73	0.92	[24]
			docetaxel 75mg/m^2^ 3wk		64	58	(0.60, 0.89)	(0.77, 1.10)	
R S Herbst	2016	1034	pembrolizumab 2 mg/kg	NSCLC	63	62	0.71	0.88	[25]
			pembrolizumab 10 mg/kg		65	62	(0.58, 0.88)	(0.74, 1.05)	
			docetaxel		62	61	0.61	0.79	
							(0.49, 0.75)	(0.66, 0.94)	
L Fehrenbacher	2016	287	atezolizumab 1200 mg	NSCLC	62	65	0.73	0.94	[26]
			docetaxel 75 mg/m^2^ 3wk		143	53	(0.53, 0.99)	(0.72, 1.23)	
J. Larkin	2015	945	nivolumab 3mg/kg 2wk	melanoma	59	63.9	0.65	0.57	[10]
			nivolumab 1mg/kg 3wk +		59	65.6	(0.39, 1.08)	(0.43, 0.76)	
			ipilimumab 3mg/kg 3wk						
			ipilimumab 3mg/kg 3wk		61	64.1			
C. Robert	2015	418	nivolumab 3mg/kg 2wk	melanoma	64	57.6	0.42	0.43	[27]
			dacarbazine 1000 mg/m^2^ 3wk		66	60.1	(0.25, 0.73)	(0.34, 0.56)	
C. Robert	2015	834	pembrolizumab 10 mg/kg 2wk	melanoma	61	57.7	0.63	0.58	[28]
			pembrolizumab 10mg/kg 3wk		63	62.8	(0.47, 0.83)	(0.46, 0.72)	
			ipilimumab 3mg/kg 3wk		62	58.3	0.69	0.58	
							(0.52, 0.90)	(0.47, 0.72)	
M A. Postow	2015	142	nivolumab 1 mg/kg+	melanoma	64	66	N/A	0.40	[29]
			ipilimumab 3mg/kg					(0.23, 0.68)	
			ipilimumab 3mg/kg		67	68		0.38	
								(0.15,1.00)	
A Ribas	2015	540	pembrolizumab 2 mg/kg 3wk	melanoma	62	58	N/A	0.57	[30]
			pembrolizumab 10mg/kg 3wk		60	60		(0.45, 0.73)	
			IC chemotherapy		63	64		0.50	
								(0.39, 0.64)	
J S Weber	2015	631	Nivolumab 3mg/kg 2wk	melanoma	59	65	N/A	0.82	[31]
			IC chemotherapy		62	64		(0.40,1.66)	
R. J. Motzer	2015	821	nivolumab 3mg/kg 2wk	RCC	62	77	0.73	0.88	[32]
			everolimus 10mg		62	74	(0.60, 0.89)	(0.75, 1.03)	

### Meta-analyses results

The data available on OS pooling were from 10 observations. The pooled HR for OS (Table 2) was 0.67, (95%CI, 0.62, 0.73; *p* < 0.001) without significant heterogeneity (I^2^ < 0.1%), which reflected that compared to non-PD-1/PD-L1 therapy, PD-1/PD-L1 blockades reduced 33% in risk of death among epithelial carcinoma patients. This benefit had met the criteria of treatment superiority.

**Table 2:** the pooled results of HRs for OS and PFS of the included trials

Outcome endpoint	Cancer Type	Subgroup	Number of observations	Publication bias (*P*>|t|)*	HR, (95%CI)	*p*	Pooling model	I^2^%
OS	All types	--	10	0.064	0.67, (0.62, 0.73)	0.000	Fixed	0.0
OS	All types	PD-L1					Fixed	
		expression≥1%	5	0.929	0.58, (0.50, 0.66)	0.000		5.6
		PD-L1	5	0.046	0.75, (0.63, 0.89)	0.001		0.0
		expression<1%						
OS	All types	Age≥65	5	0.291	0.72, (0.64, 0.82)	0.000	Fixed	0.0
		Age<65	7	0.857	0.70, (0.60, 0.81)	0.000		0.0
OS	All types	Male	5	0.366	0.68, (0.60, 0.77)	0.000	Fixed	0.0
		Female	5	0.775	0.75, (0.64, 0.88)	0.000		0.0
OS	All types	ECOG score=0	4	0.181	0.67, (0.56, 0.80)	0.000	Fixed	0.0
		ECOG score=1	4	0.829	0.69, (0.60, 0.80)	0.000		0.0
OS	NSCLC	Squamous	3	0.865	0.67, (0.54, 0.82)	0.000	Fixed	0.0
		Non-squamous	3	0.162	0.69, (0.60, 0.79)	0.000		0.0
OS	NSCLC	*EGFR* wild type	2	--	0.66, (0.57, 0.77)	0.000	Fixed	0.0
		*EGFR* mutant type	2	--	1.40, (0.92, 2.12)	0.114		67.3
OS	NSCLC	Smoker	2	--	0.71, (0.60, 0.86)	0.000	Fixed	0.0
		Never-Smoker	2	--	0.88, (0.59, 1.32)	0.536		39.0
PFS	All types	--	15	0.063	0.66, (0.57, 0.76)	0.000	Random	79.7
PFS	Melanoma	--	9	0.668	0.54, (0.49, 0.59)	0.000	Fixed	0.0
	NSCLC		5	0.488	0.84, (0.77, 0.92)	0.000		44.4
	RCC		1	--	0.84, (0.75, 1.03)	0.114		--
PFS	All types	PD-L1					Fixed	
		expression≥1%	3	0.183	0.59, (0.48, 0.72)	0.000		0.0
		PD-L1	3	0.236	0.80, (0.59, 1.07)	0.136		0.0
		expression<1%						
PFS	All types	Age≥65	5	0.050	0.57, (0.44, 0.74)	0.000	Random	77.6
		Age<65	5	0.000	0.69, (0.56, 0.84)	0.000		46.8
PFS	All types	Male	5	0.001	0.60, (0.49, 0.72)	0.000	Random	54.6
		Female	5	0.073	0.67, (0.51, 0.87)	0.002		66.3
PFS	All types	ECOG score=0	5	0.541	0.64, (0.48, 0.84)	0.001	Random	77.3
		ECOG score=1	5	0.028	0.65, (0.56, 0.75)	0.000		18.4
PFS	Melanoma	*BRAF* wild type	5	0.632	0.51, (0.45, 0.58)	0.000	Fixed	2.7
		*BRAF* mutant type	6	0.342	0.55, (0.44, 0.69)	0.000		8.0

**Figure 3:**
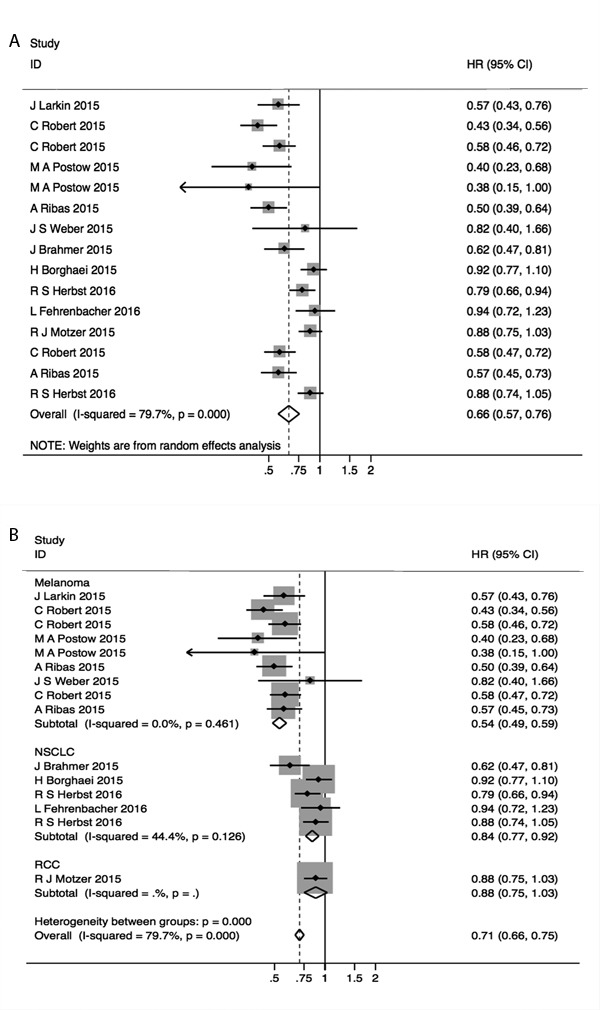
Forest plots of A. hazard ratio (HR) for PFS; B. HRs for PFS in subgroups of different types of epithelial carcinoma

All studies reported the data on PFS, and the combined HR for PFS with 15 records was 0.66, (95%CI, 0.57, 0.76; *p* < 0.001). However, a considerable heterogeneity with I^2^ = 79.7 % was observed with the random effect model (Figure 3A). Hence, we conducted the subgroup analyses to investigate the cause of heterogeneity and divided the studies into different cancer types (melanoma, NSCLC and RRC). The results of different types analyses had moderate within-group heterogeneities with I^2^ < 0.1% for melanoma and 44% for NSCLC. The computed HRs (95%CI; p) for PFS in melanoma, NSCLC and RCC were 0.54 (0.49, 0.59; *p* < 0.001), 0.84 (0.77, 0.92; *p* < 0.001), 0.84 (0.75, 1.03; *p* = 0.114) respectively.

**Figure 4:**
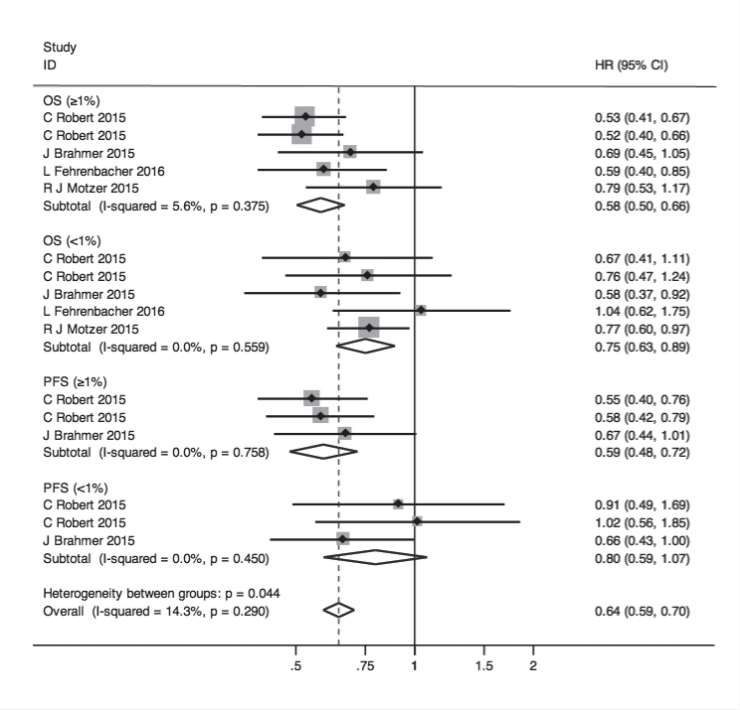
Forest plots of HRs for OS and PFS in the subgroups of patients with PD-L1 expression ≥ 1% and < 1%

To investigate predictive role of PD-L1 expression, we computed the aggregated HRs for OS and PFS in different levels of PD-L1 expression ( ≥ 1%, < 1%) groups and generated the forest plot (Figure 4). Pooled HRs (95%CI; p) were 0.58, (0.50, 0.66; *p* < 0.001) in PD-L1 ≥ 1% group, 0.75, (0.63, 0.89; *p* = 0.001) in PD-L1 < 1% group for OS and 0.59, (0.48, 0.72; *p* < 0.001) in PD-L1 ≥ 1% group, 0.80, (0.59, 1.07; *p* = 0.136) in PD-L1 < 1% group for PFS with not important heterogeneity within all subgroups (I^2^ = 5.6%,0.0%,0.0%,0.0%).

We also analyzed the baseline data such as age, sex and ECOG score of 0 or 1 (on a 5-point scale, with higher scores indicating greater disability) to further explore other factors. The results of pooled HRs for OS and PFS corresponding to these factors were listed in Table 2 and the effects of anti-PD-1/PD-L1 therapy in all those subgroups were favorable and did not have significantly changes between different ages, sexes and ECOG scores groups.

**Figure 5:**
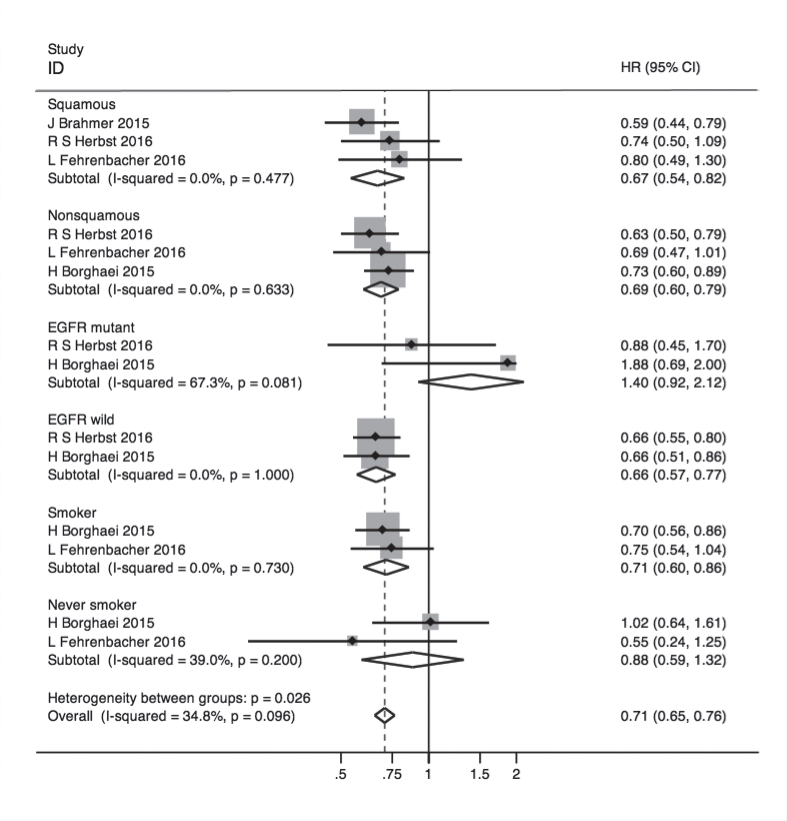
Forest plots of HRs for OS in squamous, non-squamous, *EGFR* mutant, *EGFR* wild, Smoker and never smoker NSCLC subgroup patients

Among the NSCLC patients, both the squamous patients (HR (95%CI) = 0.67, (0.54, 0.82); *p* < 0.001) and non-squamous patients (0.69, (0.60, 0.79); *p* < 0.001) had more extended OS when compared with non-PD-1/PD-L1 therapy. In *EGFR* wild type patients, the HR for OS was 0.66, (0.57, 0.77); *p* < 0.001, indicating the better efficacy of PD-1/PD-L1 blockades in those patients. However, in patients with *EGFR* mutant type, the HR for OS was 1.40, (0.92, 2.12) and p was 0.114 (I^2^of heterogeneity = 67.3%), suggesting that anti-PD-1/PD-L1 therapy functioned not distinctly better than the control group treatment. Similarly, the never-smokers were verified not to have the expectedly longer OS with HR pooled as 0.88, (0.59, 1.32); *p* = 0.536 with I^2^ = 39.0%. The HR for OS in smokers was 0.71, (0.60, 0.86); *p* < 0.001 with the meaning of better outcomes due to the use of anti-PD-1/PD-L1 agents. Besides, in melanoma patients, both the *BRAF* wild type and *BRAF* mutant patients benefited in OS, and HRs (95%CIs) were 0.51, (0.45, 0.58), *p* < 0.001; 0.55, (0.44, 0.69), *p* < 0.001 respectively with not important heterogeneity (I^2^ = 2.7%; I^2^ = 8.0%) (Figure 5).

All reported data of the meta-analyses results were listed in Table 2 with the relative details and models adopted.

### Sensitivity analyses

The sensitivity analyses were conducted by excluding studies one by one. The results of those analyses showed no significant differences when compared to the former summary estimates and had excellent stability.

### Publication bias

We assessed the publication bias of included data for pooled analyses using the Egger's test, and the p values were listed in Table 2. In the light of data computed and listed, there was no substantial publication bias (*p* < 0.05) in our main analyses. Nevertheless, two possible biases with *p* < 0.05 by Egger's test were observed in the subgroups of Age < 65 and Male patients in the pooling process of HR for PFS.

## DISCUSSION

It was acknowledged that PD-1/PD-L1 blockade, as a type of immune checkpoint inhibitors, had remarkable response rate and clinical results in patients with different kinds of cancer, especially those with epithelial-originated malignancies. Nonetheless, it remained unclear whether anti-PD-1/PD-L1 therapy, in contrast with other therapy, functioned better to extend OS and PFS and which subgroups of patients would benefit from the treatment. Our meta-analysis integrating all data from relevant trials were requested to solve the problem. To our knowledge, the meta-analysis was the first study to investigate the outcome and predictive biomarkers for PD-1/PD-L1 therapy in epithelial carcinoma patients within solely randomized controlled trials by assessing the two primary endpoints of OS and PFS.

Our results provided convincing evidence that the OS of patients given PD-1/PD-L1 inhibitors was significantly longer than patients given other drugs as those individual studies reported (only one study comparing nivolumab with ipilimumab (CTLA-4 antibody) [10] excluded). The effect of PD-1/PD-L1 inhibitors on prolonging PFS was controversial among the 15 observations from 11 enrolled trials and our aggregated HR affirmed the efficacy to extend PFS in epithelial carcinoma patients. However, the heterogeneity of the studies was significant and we conducted the subgroup analyses stratified by different cancer types to figure out this issue. The results of subgroup analyses indicated that both the melanoma and NSCLC patients could obtain longer PFS due to the use of PD-1/PD-L1 blockades. Whilst HR for PFS in one RCC trial suggested that no difference in PFS between the two interventions existed.

In PD-L1 expression investigation, we found it not practicable to use PD-L1 as a biomarker to predict OS benefit when comparing PD-1/PD-L1 blockades with other control therapy, for both the higher (PD-L1 expression ≥ 1%) and lower (PD-L1 expression < 1%) expression groups could gain obvious clinical benefit from PD-1/PD-L1 blockades. Be that as it may, the higher expression group had the better PFS outcome, but the lower expression group was associated with insubstantial improvement of PFS. The immunohistochemistry (IHC) cut-off value (ranging from 1% to 50%) we chose to define the PD-L1 positivity was 1%, in that the clinical data assessed by this point were the most abundant in the included 11 trials. Besides, cutting off by the lowest expression level enabled to include the most possible patients who could benefit from the therapy. However, the assessment of PD-L1 expression was much complicated because the tumor PD-L1 expression was not constant which was associated with activated tumor antigen-specific T cells [11, 12] and could be induced by specific agents such as interferon [13]. In addition, the degree of PD-L1 expression could be heterogeneous between different types of cancer or even primary and metastatic lesions in one type of cancer [12]. As for detection methods, there were still many limitations of IHC detection such as the low efficacy caused by the two small hydrophilic regions of PD-L1 antibody and the bias caused by different proprietary assays in different trials [14, 15]. Several associations were focusing on standardizing and validating a reliable IHC assay of PD-L1 expression currently [16]. The combination of IHC and gene amplification as the detection method adopted in HER-2 status assessment in gastric and breast cancers [17, 18] also gave the instruction for PD-L1 detection [19, 20]. Thus, the further experiments focused on the expression mechanism and detection were needed to draw more definitive conclusion.

The next novel finding of our analyses was in different subgroups. We found that the *BRAF* mutant or *BRAF* wild type patients in melanoma group, and the squamous cancer patient or non-squamous cancer patients in NSCLC group had gained better outcome of survival. But there was no significant improvement for OS in patients without smoking history and patients with *EGFR* mutations. Whilst, the present or previous smoker, patients with *EGFR* wild type showed longer survival time, implicating the smoking history and *EGFR* wild type might be considered as the potential predictive factors for anti-PD-1/PD-L1 treatment. In our speculation, the association of mutations and other exposures to mutagens like smoking with the efficacy of PD-L1 blockades was possibly because the tumor antigen, considered as the target of T cell activated by checkpoint blockade, was related to the consequence of somatic mutations [19, 21, 22]. However, the limited number of observations in our subgroup analyses still required prospective validation with larger scale investigations.

Finally, OS had been improved in the overall patient regardless of the age, sex and ECOG score. Thus, it was persuasive that those factors were not meaningful indicators for the eligibility of anti-PD-1/PD-L1 treatment.

In conclusion, the aggregated HRs for OS and PFS summarized in our systematic analyses revealed that in the comparison of anti-PD-1/PD-L1 agents with other control therapy, the PD-L1 expression was not an appropriate factor to predict the benefit of OS in epithelial carcinoma patients, but could be predictive for PFS. Age, sex and ECOG score were excluded to predict any of the outcome endpoints. Smoking history and *EGFR* wild type were potential indicators for prolonged OS in NSCLC patients. There were multiple clinical trials ongoing and many other antibodies targeting PD-1/PD-L1 under early-stage development currently, more comprehensive data from future clinical trials focused on this field were still needed for further investigation.

## MATERIALS AND METHODS

### Publications search

We searched for the articles of clinical trials from PubMed, Embase, Web of Science, and the Cochrane Library from January 2010 to April 2016. Records at American Society of Clinical Oncology (ASCO), the European Society for Medical Oncology (ESMO), the world Conference of Lung cancer (WCLC) were also reviewed. The following search terms were used: “pembrolizumab”, “Nivolumab” “atezolizumab”, “Tremelimumab”, “AMP-224”, “MDX-1105”, “pidilizumab”, and “cancer/carcinoma”.

### Study selection

All relevant articles underwent evaluation for eligibility by two investigators independently and we selected the articles according to the following criteria: 1) articles with randomized controlled trials (RCTs); 2) at least one of the two endpoints (PFS, OS) reported; 3) published in English; 4) the full text available. Our exclusion criteria were as below: 1) letters, expert opinions, case reports and reviews; 2) articles without available data; 3) duplicate publications.

### Quality assessment

We assessed the quality of involved randomized controlled clinical trials according to the criteria presented in the *Cochrane Handbook for Systematic Reviews of Interventions* (version 5.1.0; chapter 8), and evaluated the random sequence generation, allocation concealment, blinding of participants and personnel, blinding of outcome assessment, incomplete outcome data, selective reporting and other bias to ensure the low-risk of bias of the studies included.

### Data extraction

The data of study identification, the intervention of experimental treatment and control group, numbers of enrolled patients in each trial, patients' detailed information (age, sex, line of therapy and ECOG score), hazard ratios(HR) with their 95%CIs and p values for OS and PFS were extracted by two individual investigators independently. We also collected the relevant information in every subgroup we set to render sufficient data to our subgroup analyses.

### Statistical analysis

We calculated pooled HRs and their 95%CIs for OS and PFS which were considered to be the primary outcome of the meta-analyses and generated the forest plots accordingly. The chi-square Q test and I^2^ statistic were used to indicate the heterogeneity. A p value less than 0.05 in the Q test or an I^2^ value greater than 50% in the I^2^ statistics suggested the significant heterogeneity. If the heterogeneity was significant, we used the random effect model of pooling instead of fixed effect model and designed subgroups analyses to clarify the between-study heterogeneity. To test the publication bias of the included studies, the Egger's test was chosen. All the statistical analyses were performed with STATA/SE software version12.0 (STATA Corporation, College Station, TX, USA).

## References

[1] Couzin-Frankel J (2013). Breakthrough of the year 2013. Cancer immunotherapy. Science (New York, NY).

[2] Postow MA, Callahan MK, Wolchok JD (2015). Immune Checkpoint Blockade in Cancer Therapy. Journal of clinical oncology.

[3] Chow LQ (2013). Exploring novel immune-related toxicities and endpoints with immune-checkpoint inhibitors in non-small cell lung cancer.

[4] Bagley SJ, Bauml JM, Langer CJ (2015). PD-1/PD-L1 immune checkpoint blockade in non-small cell lung cancer. Clinical advances in hematology & oncology.

[5] Gandini S, Massi D, Mandala M (2016). PD-L1 expression in cancer patients receiving anti PD-1/PD-L1 antibodies: A systematic review and meta-analysis. Critical reviews in oncology/hematology.

[6] Rosenberg JE, Hoffman-Censits J, Powles T, van der Heijden MS, Balar AV, Necchi A, Dawson N, O'Donnell PH, Balmanoukian A, Loriot Y, Srinivas S, Retz MM, Grivas P, Joseph RW, Galsky MD, Fleming MT (2016). Atezolizumab in patients with locally advanced and metastatic urothelial carcinoma who have progressed following treatment with platinum-based chemotherapy: a single-arm, multicentre, phase 2 trial. Lancet (London, England).

[7] Passiglia F, Bronte G, Bazan V, Natoli C, Rizzo S, Galvano A, Listi A, Cicero G, Rolfo C, Santini D, Russo A (2016). PD-L1 expression as predictive biomarker in patients with NSCLC: a pooled analysis. Oncotarget.

[8] Taube JM, Klein A, Brahmer JR, Xu H, Pan X, Kim JH, Chen L, Pardoll DM, Topalian SL, Anders RA (2014). Association of PD-1, PD-1 ligands, and other features of the tumor immune microenvironment with response to anti-PD-1 therapy. Clinical cancer research.

[9] Tumeh PC, Harview CL, Yearley JH, Shintaku IP, Taylor EJ, Robert L, Chmielowski B, Spasic M, Henry G, Ciobanu V, West AN, Carmona M, Kivork C, Seja E, Cherry G, Gutierrez AJ (2014). PD-1 blockade induces responses by inhibiting adaptive immune resistance. Nature.

[10] Larkin J, Chiarion-Sileni V, Gonzalez R, Grob JJ, Cowey CL, Lao CD, Schadendorf D, Dummer R, Smylie M, Rutkowski P, Ferrucci PF, Hill A, Wagstaff J, Carlino MS, Haanen JB, Maio M (2015). Combined Nivolumab and Ipilimumab or Monotherapy in Untreated Melanoma. The New England journal of medicine.

[11] Taube JM, Anders RA, Young GD, Xu H, Sharma R, McMiller TL, Chen S, Klein AP, Pardoll DM, Topalian SL, Chen L (2012). Colocalization of inflammatory response with B7-h1 expression in human melanocytic lesions supports an adaptive resistance mechanism of immune escape. Science translational medicine.

[12] Madore J, Vilain RE, Menzies AM, Kakavand H, Wilmott JS, Hyman J, Yearley JH, Kefford RF, Thompson JF, Long GV, Hersey P, Scolyer RA (2015). PD-L1 expression in melanoma shows marked heterogeneity within and between patients: implications for anti-PD-1/PD-L1 clinical trials. Pigment cell & melanoma research.

[13] Lee SJ, Jang BC, Lee SW, Yang YI, Suh SI, Park YM, Oh S, Shin JG, Yao S, Chen L, Choi IH (2006). Interferon regulatory factor-1 is prerequisite to the constitutive expression and IFN-gamma-induced upregulation of B7-H1 (CD274). FEBS letters.

[14] Dong H, Zhu G, Tamada K, Chen L (1999). B7-H1, a third member of the B7 family, co-stimulates T-cell proliferation and interleukin-10 secretion. Nature medicine.

[15] Sznol M, Chen L (2013). Antagonist antibodies to PD-1 and B7-H1 (PD-L1) in the treatment of advanced human cancer. Clinical cancer research.

[16] Kerr KM, Tsao MS, Nicholson AG, Yatabe Y, Wistuba II, Hirsch FR (2015). Programmed Death-Ligand 1 Immunohistochemistry in Lung Cancer: In what state is this art?. Journal of thoracic oncology.

[17] Kaptain S, Tan LK, Chen B (2001). Her-2/neu and breast cancer. Diagnostic molecular pathology.

[18] Bang YJ, Van Cutsem E, Feyereislova A, Chung HC, Shen L, Sawaki A, Lordick F, Ohtsu A, Omuro Y, Satoh T, Aprile G, Kulikov E, Hill J, Lehle M, Ruschoff J, Kang YK (2010). Trastuzumab in combination with chemotherapy *versus* chemotherapy alone for treatment of HER2-positive advanced gastric or gastro-oesophageal junction cancer (ToGA): a phase 3, open-label, randomised controlled trial. Lancet (London, England).

[19] Meng X, Huang Z, Teng F, Xing L, Yu J (2015). Predictive biomarkers in PD-1/PD-L1 checkpoint blockade immunotherapy. Cancer treatment reviews.

[20] Sabatier R, Finetti P, Mamessier E, Adelaide J, Chaffanet M, Ali HR, Viens P, Caldas C, Birnbaum D, Bertucci F (2015). Prognostic and predictive value of PDL1 expression in breast cancer. Oncotarget.

[21] Rizvi NA, Hellmann MD, Snyder A, Kvistborg P, Makarov V, Havel JJ, Lee W, Yuan J, Wong P, Ho TS, Miller ML, Rekhtman N, Moreira AL, Ibrahim F, Bruggeman C, Gasmi B (2015). Cancer immunology. Mutational landscape determines sensitivity to PD-1 blockade in non-small cell lung cancer. Science (New York, NY).

[22] Gubin MM, Zhang X, Schuster H, Caron E, Ward JP, Noguchi T, Ivanova Y, Hundal J, Arthur CD, Krebber WJ, Mulder GE, Toebes M, Vesely MD, Lam SS, Korman AJ, Allison JP (2014). Checkpoint blockade cancer immunotherapy targets tumour-specific mutant antigens. Nature.

[23] Brahmer J, Reckamp KL, Baas P, Crino L, Eberhardt WE, Poddubskaya E, Antonia S, Pluzanski A, Vokes EE, Holgado E, Waterhouse D, Ready N, Gainor J, Aren Frontera O, Havel L, Steins M (2015). Nivolumab *versus* Docetaxel in Advanced Squamous-Cell Non-Small-Cell Lung Cancer. The New England journal of medicine.

[24] Borghaei H, Paz-Ares L, Horn L, Spigel DR, Steins M, Ready NE, Chow LQ, Vokes EE, Felip E, Holgado E, Barlesi F, Kohlhaufl M, Arrieta O, Burgio MA, Fayette J, Lena H (2015). Nivolumab *versus* Docetaxel in Advanced Nonsquamous Non-Small-Cell Lung Cancer. The New England journal of medicine.

[25] Herbst RS, Baas P, Kim DW, Felip E, Perez-Gracia JL, Han JY, Molina J, Kim JH, Arvis CD, Ahn MJ, Majem M, Fidler MJ, de Castro G, Garrido M, Lubiniecki GM, Shentu Y (2016). Pembrolizumab *versus* docetaxel for previously treated, PD-L1-positive, advanced non-small-cell lung cancer (KEYNOTE-010): a randomised controlled trial. Lancet (London, England).

[26] Fehrenbacher L, Spira A, Ballinger M, Kowanetz M, Vansteenkiste J, Mazieres J, Park K, Smith D, Artal-Cortes A, Lewanski C, Braiteh F, Waterkamp D, He P, Zou W, Chen DS, Yi J (2016). Atezolizumab *versus* docetaxel for patients with previously treated non-small-cell lung cancer (POPLAR): a multicentre, open-label, phase 2 randomised controlled trial. Lancet (London, England).

[27] Robert C, Long GV, Brady B, Dutriaux C, Maio M, Mortier L, Hassel JC, Rutkowski P, McNeil C, Kalinka-Warzocha E, Savage KJ, Hernberg MM, Lebbe C, Charles J, Mihalcioiu C, Chiarion-Sileni V (2015). Nivolumab in previously untreated melanoma without BRAF mutation. The New England journal of medicine.

[28] Robert C, Schachter J, Long GV, Arance A, Grob JJ, Mortier L, Daud A, Carlino MS, McNeil C, Lotem M, Larkin J, Lorigan P, Neyns B, Blank CU, Hamid O, Mateus C (2015). Pembrolizumab *versus* Ipilimumab in Advanced Melanoma. The New England journal of medicine.

[29] Postow MA, Chesney J, Pavlick AC, Robert C, Grossmann K, McDermott D, Linette GP, Meyer N, Giguere JK, Agarwala SS, Shaheen M, Ernstoff MS, Minor D, Salama AK, Taylor M, Ott PA (2015). Nivolumab and ipilimumab *versus* ipilimumab in untreated melanoma. The New England journal of medicine.

[30] Ribas A, Puzanov I, Dummer R, Schadendorf D, Hamid O, Robert C, Hodi FS, Schachter J, Pavlick AC, Lewis KD, Cranmer LD, Blank CU, O'Day SJ, Ascierto PA, Salama AK, Margolin KA (2015). Pembrolizumab *versus* investigator-choice chemotherapy for ipilimumab-refractory melanoma (KEYNOTE-002): a randomised, controlled, phase 2 trial. The Lancet Oncology.

[31] Weber JS, D'Angelo SP, Minor D, Hodi FS, Gutzmer R, Neyns B, Hoeller C, Khushalani NI, Miller WH, Lao CD, Linette GP, Thomas L, Lorigan P, Grossmann KF, Hassel JC, Maio M (2015). Nivolumab *versus* chemotherapy in patients with advanced melanoma who progressed after anti-CTLA-4 treatment (CheckMate 037): a randomised, controlled, open-label, phase 3 trial. The Lancet Oncology.

[32] Motzer RJ, Escudier B, McDermott DF, George S, Hammers HJ, Srinivas S, Tykodi SS, Sosman JA, Procopio G, Plimack ER, Castellano D, Choueiri TK, Gurney H, Donskov F, Bono P, Wagstaff J (2015). Nivolumab *versus* Everolimus in Advanced Renal-Cell Carcinoma. The New England journal of medicine.

